# Antisense Transcription across Nucleotide Repeat Expansions in Neurodegenerative and Neuromuscular Diseases: Progress and Mysteries

**DOI:** 10.3390/genes11121418

**Published:** 2020-11-27

**Authors:** Ana F. Castro, Joana R. Loureiro, José Bessa, Isabel Silveira

**Affiliations:** 1 Genetics of Cognitive Dysfunction Laboratory, i3S- Instituto de Investigação e Inovação em Saúde, Universidade do Porto, 4200-135 Porto, Portugal; afcastro@i3s.up.pt (A.F.C.); joana.loureiro@ibmc.up.pt (J.R.L.); 2 IBMC-Institute for Molecular and Cell Biology, Universidade do Porto, 4200-135 Porto, Portugal; jose.bessa@ibmc.up.pt; 3 ICBAS, Universidade do Porto, 4050-313 Porto, Portugal; 4 Vertebrate Development and Regeneration Laboratory, i3S- Instituto de Investigação e Inovação em Saúde, Universidade do Porto, 4200-135 Porto, Portugal

**Keywords:** splicing misregulation, RNA foci, trinucleotide repeats, nuclear inclusions

## Abstract

Unstable repeat expansions and insertions cause more than 30 neurodegenerative and neuromuscular diseases. Remarkably, bidirectional transcription of repeat expansions has been identified in at least 14 of these diseases. More remarkably, a growing number of studies has been showing that both sense and antisense repeat RNAs are able to dysregulate important cellular pathways, contributing together to the observed clinical phenotype. Notably, antisense repeat RNAs from spinocerebellar ataxia type 7, myotonic dystrophy type 1, Huntington’s disease and frontotemporal dementia/amyotrophic lateral sclerosis associated genes have been implicated in transcriptional regulation of sense gene expression, acting either at a transcriptional or posttranscriptional level. The recent evidence that antisense repeat RNAs could modulate gene expression broadens our understanding of the pathogenic pathways and adds more complexity to the development of therapeutic strategies for these disorders. In this review, we cover the amazing progress made in the understanding of the pathogenic mechanisms associated with repeat expansion neurodegenerative and neuromuscular diseases with a focus on the impact of antisense repeat transcription in the development of efficient therapies.

## 1. Introduction

**Figure 1:**
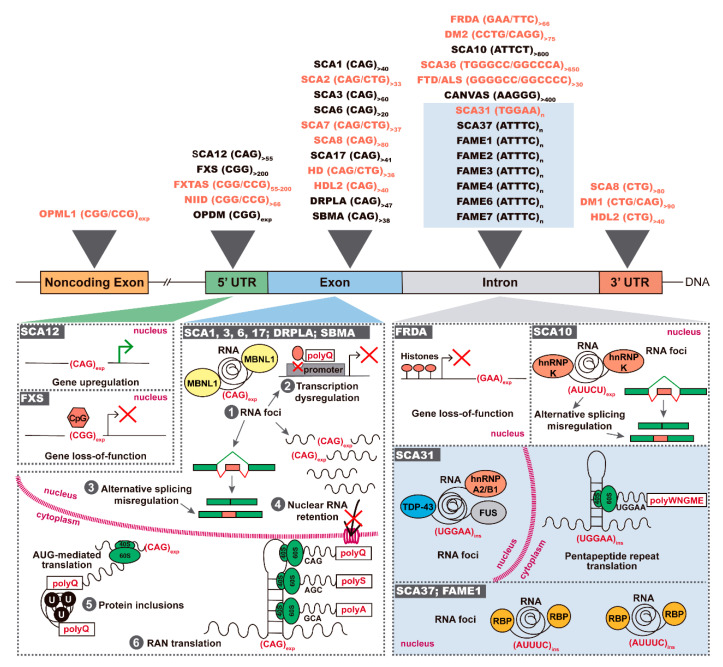
Repeat expansions and insertions causing neurodegenerative and neuromuscular diseases. Top: pathogenic repeat expansions spread over 5′ and 3′ UTRs, exons and introns; novel repeat insertions are highlighted in a blue box; bidirectional transcription is known for the disorders in orange. Bottom: in SCA12, a 5′UTR (CAG)_n_ expansion leads to gene upregulation, whereas expanded (CGG)_n_ leads to CpG hypermethylation with silencing of FMRP expression in FXS; coding transcripts containing the expanded (CAG)_n_ are able to (1) sequester RBPs such as MBNL1, forming nuclear RNA foci, which causes dysregulation of several cellular processes like (2) transcription, (3) mRNA splicing, (4) nucleocytoplasmic transport; in the cytoplasm, coding repeat expansions are (5) translated in proteins with expanded polyQ tracts leading to ubiquitin-positive (U) inclusions in neurons, or are (6) RAN-translated in polypeptides; in FRDA, a biallelic intronic (GAA)_n_ expansion leads to repressive chromatin with consequent gene silencing; in SCA10, the (ATTCT)_n_ is transcribed forming nuclear RNA foci with hnRNP K; in SCA31, nuclear RNA foci colocalize with TDP-43, FUS and hnRNP A2/B1 and pentapeptides are produced. PolyQ-polyglutamine; polyS-polyserine; polyA-polyalanine; polyWNGME-poly-tryptophan-asparagine-glycine-methionine-glutamic acid.

In the last few decades, an increasing number of neurological and neuromuscular diseases has been associated with expansions of microsatellites in coding and noncoding gene regions. Microsatellites are polymorphic repeat sequences of 1 to 9 nucleotides distributed throughout the genome, composing about 3% of the human genome. These polymorphic repeats are usually pathogenic when they expand in size above a given threshold [1,2]. In 1991, the discovery of the first repeat expansions overlapped with the finding of the causative genes for fragile X syndrome (FXS) and spinal bulbar muscular atrophy [3,4]. Since then, more than 30 neurodegenerative and neuromuscular diseases originated by expansions of tri-, tetra-, penta- or hexanucleotide repeats have been molecularly identified [1,2] (Figure 1). Trinucleotide repeat expansions are located in coding gene regions or in noncoding gene regions, whereas pathogenic tetra-, penta- and hexanucleotide repeats have only been found in noncoding gene regions [5]. More recently, a new type of pathogenic repeats has been identified, consisting in the insertion of a new unstable pentanucleotide repeat within a pre-existent microsatellite. These pentanucleotide repeat insertions have been found in spinocerebellar ataxia type 31 (SCA31) [6], SCA37 [7] and six types of familial adult myoclonic epilepsy (FAME 1, 2, 3, 4, 6 and 7) [8,9,10,11] (Figure 1).

Depending on the repeat motif and its location in a gene, three different pathogenic mechanisms have been identified in repeat expansion diseases, consisting in protein gain-of-function, gene loss-of-function and/or RNA gain-of-function [1] (Figure 1). In coding regions, trinucleotide expansions of CAG and GCN repeats encode toxic stretches of polyglutamine (polyQ) or polyalanine (polyA), respectively. The proteins containing the expanded polyQ are misfolded and gain the aberrant ability to recruit other cellular proteins, forming toxic ubiquitin-positive aggregates in the cell cytoplasm and/or nucleus. The proteins sequestered in polyQ aggregates are then unavailable in the cell to perform their native function, dysregulating several molecular pathways [2,3]. In noncoding gene regions, the expanded repeats may trigger gene silencing or RNA-mediated toxicity. If the expanded repeat leads to modifications of the chromatin state through methylation of CpGs or histones, there is decrease or loss of gene transcription and, consequently, a reduction of gene encoded protein. In contrast, when the expanded repeat is transcribed, the RNA sequesters RNA-binding proteins (RBPs), forming nuclear RNA aggregates. Consequently, these RBPs do not play their function normally in the cell, impairing several cellular processes like splicing and nucleocytoplasmic transport [1,4]. In the cytoplasm, these expanded repeat RNAs may trigger repeat-associated non-ATG (RAN) translation because they are able to recruit ribosomal subunits initiating repeat translation across the three possible reading frames, generating homopolymeric, di-, tetra- or penta- repeat peptides [1,5,6,7] (Table 1).

**Figure 2:**
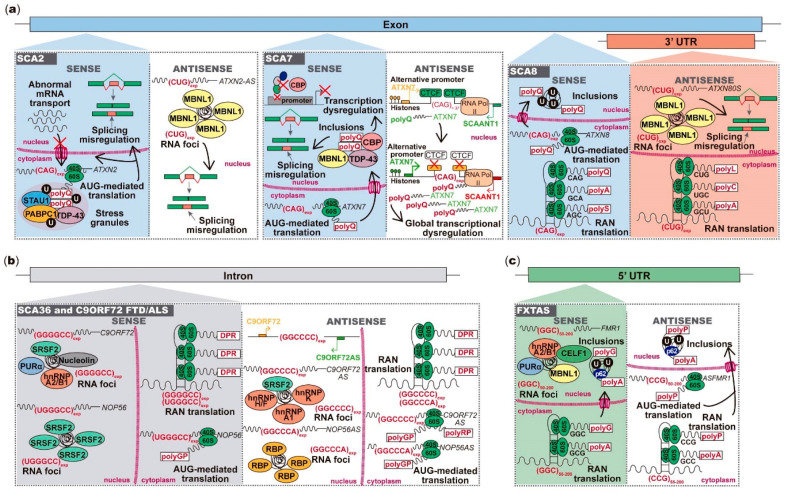

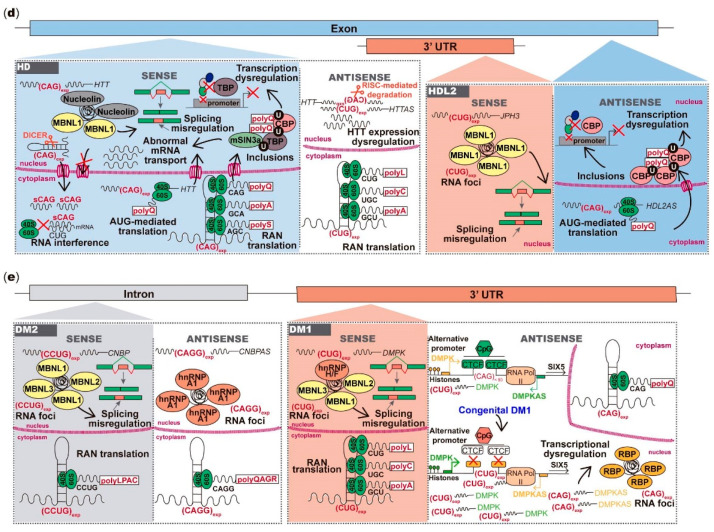
Toxicity mediated by microsatellite repeats bidirectionally transcribed in neurodegenerative and neuromuscular diseases. (**a**) The (CAG)_exp_ RNA from *ATXN2* is translated into polyQ that abnormally interact with proteins in stress granules; the *ATXN2-AS* (CUG)_exp_ forms RNA foci with splicing factors leading to misplicing of other mRNAs; from the *ATXN7* strand, the (CAG)_exp_ RNA is translated into toxic polyQ that form nuclear inclusions with RNA and DNA-binding proteins, dysregulating transcription and cellular mRNAs splicing; in *ATXN7AS*, the (CUG)_exp_ reduces CTCF-binding, causing *SCAANT1* downregulation and consequent derepression of *ATXN7* promoter, leading to ataxin-7 overexpression and global transcriptional dysregulation; from the *ATXN8* strand, the (CAG)_exp_ RNA is translated into toxic polyQ that form ubiquitin-positive intranuclear inclusions; the *ATXN8OS* (CUG)_exp_ forms nuclear RNA foci causing misplicing or is RAN-translated. (**b**) In SCA36 and *C9ORF72* FTD/ALS, sense and antisense strand RNAs aggregate in nuclear RNA foci or are translated into DRPs. (**c**) The *FMR1* (GGC)_55–200_ RNA forms nuclear foci or is RAN-translated into toxic peptides, which form intranuclear inclusions; from *ASFMR1* strand, the (GCC)_55–200_ RNA can be translated into toxic peptides, leading to intranuclear aggregation. (**d**) From the *HTT* strand, the (CAG)_exp_ RNA can aggregate in nuclear RNA foci or form hairpins posteriorly cleaved by DICER; the (CAG)_exp_ RNA is also translated into polyQ proteins, forming nuclear inclusions, or it is RAN-translated; in *HTTAS* orientation, the (CUG)_exp_ RNA regulates the *HTT* RNA on a RISC-dependent manner and can be RAN-translated into polypeptides. In HDL2, the (CAG)_exp_ RNA forms RNA foci, leading to splicing misregulation; polyQ can be generated from the antisense (CAG)_exp_ RNA, impairing the cellular transcriptional activity. (**e**) In DM2, both sense and antisense repeat RNAs form foci or are RAN-translated in polytetrapeptides; in DM1 *DMPK* orientation, the (CUG)_exp_ forms RNA foci with consequent misplicing of several cellular mRNAs. In congenital DM1, the (CTG)_>1000_ reduces CTCF-binding and CpG methylation, downregulating antisense transcription; antisense transcription causes *DMPK* alternative promoter upregulation, leading to DMPK overexpression; *DMPKAS* CAGs also form RNA foci and are RAN-translated. DRP-dipeptide repeats; polyQ-polyglutamine; polyA-polyalanine; polyS-polyserine; polyL-polyleucine; polyC-polycysteine; polyP-polyproline; polyG-polyglycine; polyGP-poly-glycine-proline; polyPR-poly-proline-arginine; polyLPAC-poly-leucine-proline-alanine-cysteine; polyQAGR-poly-glutamine-alanine-glycine-arginine.

Interestingly, in addition to being transcribed in gene orientation, many repeat expansions are also transcribed from the gene opposite DNA strand (Figure 2). Repeat expansion bidirectional transcription has first been identified in myotonic dystrophy type 1 (DM1) [8] followed by SCA8 [9]. Then, the number of neurological and neuromuscular diseases found with bidirectional transcription rapidly increased to include SCA2 [10], SCA7 [11], SCA31 [12], SCA36 [13,14], Fragile X-associated tremor/ataxia syndrome (FXTAS) [15], *C9ORF72* frontotemporal dementia (FTD)/amyotrophic lateral sclerosis (ALS) [16], Huntington’s disease (HD) [17], Huntington’s disease-like 2 (HDL2) [18], DM2 [6] and Friedreich ataxia (FRDA) [19] (Figure 1). More recently, sequencing reads from RNA generated from both DNA strands have been found for neuronal intranuclear inclusion disease (NIID) [20] and oculopharyngeal myopathy and leukoencephalopathy 1 (OPML1) [21] (Figure 1). Similar to the expanded repeats expressed in gene orientation, abnormal repeats transcribed from the antisense strand also have the potential to be crucial partners in the clinical manifestation of these diseases. Thus, in this review, we will cover the astonishing progress in repeat expansion disorders with a focus on bidirectional transcription of expanded repeats and its role in disease.

## 2. Antisense Expression of Trinucleotide and Pentanucleotide Repeats in Spinocerebellar Ataxias

Spinocerebellar ataxias are a genetically heterogeneous group of usually late-onset autosomal-dominant neurodegenerative diseases, characterized by progressive cerebellar ataxia due to Purkinje cell degeneration [22]. The estimated prevalence of SCAs varies significantly in different geographic regions from 1.6 to 5.6 per 100,000 inhabitants [23,24]. There are more than 40 types and at least 13 are caused by unstable repeats [22,25] (Figure 1). Here, we will cover the SCAs that have shown antisense transcript expression across the repeat expansion region, like SCA2 [10], SCA7 [11], SCA8 [9] and SCA31 [12] (Figure 2).

### 2.1. ATXN2 Sense and Antisense Trinucleotide Repeats in SCA2

SCA2 is one of the most common SCAs worldwide [24,26,27,28,29]. SCA2 is caused by a (CAG)_n_ expansion of over 33 repeat units in exon 1 of the *ATXN2* gene, which is translated into an expanded polyQ tract at the N-terminal of ataxin-2 protein [30,31,32] (Figure 1). Considering that ataxin-2 is expressed in cerebellar Purkinje cells [33,34], several mouse models have been developed to investigate the pathogenic (CAG)_>33_-mediated mechanism. The first mouse model with disrupted *Atxn2* showed motor dysfunction in the rotarod test, but no evidence of neuronal cell loss or morphologic abnormalities in the cerebellum. This suggested that *Atxn2* loss-of-function causes motor impairment, but it is not enough to cause the complete SCA2 phenotype [35]. In contrast, a transgenic mouse expressing the human full-length *ATXN2* cDNA with a (CAG)_58_ expansion, in Purkinje cells, showed motor deficits, progressive loss of dendritic arborization, cell loss and cytoplasmic ataxin-2 aggregates [36]. To better replicate SCA2 in vivo, another mouse has been generated expressing the human full-length *ATXN2* cDNA with a larger expansion, (CAG)_127_, in Purkinje cells, which presented earlier motor dysfunction accompanied by a significant decrease in expression of Purkinje cell genes such as *calbindin 28K*, indicating that an expanded ataxin-2 gain-of-function is involved in SCA2 [37]. 

Ataxin-2 is expressed in the endoplasmic reticulum and Golgi complex, playing a role in RNA metabolism and endocytosis [38,39]. Several studies have detected ataxin-2 protein binding to the 3′ terminal poly(A) of mRNAs through polyadenylate-binding protein 1 (PABPC1) interaction, allowing the initiation of translation by ribosomes [40,41]. The presence of an expanded polyQ tract would probably impair the ataxin-2 role in translation and consequently cause translational dysfunction in SCA2 [41]. Taking into account the observed gene expression dysregulation in mice and ataxin-2 function in translation, the generation of a BAC-transgenic mouse with the human *ATXN2* containing (CAG)_82_ under its endogenous promoter corroborated the decreased expression of Purkinje cell-specific genes. These mice have shown decreased cerebellar mRNA and protein levels of the highly expressed regulator of G-protein signaling 8 (RGS8), supporting ataxin-2 function in mRNA stabilization and translation [42]. These SCA2 BAC-transgenic mice also presented the characteristic Purkinje cell degeneration and progressive motor dysfunction seen in SCA2 individuals [42]. Moreover, in agreement with the Sca2 knockin mouse model carrying (CAG)_42_ [35], a decrease in *ATXN2* mRNA and protein was detected in the SCA2 BAC-transgenic mice, contrarily to BAC-transgenic mice expressing (CAG)_22_ [42]_._

Considering that ataxin-2 is key for the assembly of stress granules and P-bodies, which are important structures involved in mRNA transport, splicing and degradation in the cytoplasm, the expanded ataxin-2 interacts abnormally with several RBPs in these granules, like Staufen 1 (STAU1), Fox-1/ataxin-2-binding protein 1 (FOX/A2BP1) and DEAD-Box helicase 6 (DDX6), which suggested that these cellular processes are also dysregulated in SCA2 [43,44,45] (Table 1). In fact, in SCA2 subjects and *Atxn2* (CAG)_42_ knockin mice Purkinje cells, the expanded ataxin-2 colocalizes with STAU1 and PABPC1 proteins, respectively, into ubiquitin-positive cytoplasm granules, further corroborating an mRNA processing dysregulation in SCA2 [40,43]. Remarkably, ataxin-2 and TAR DNA-binding protein 43 (TDP-43) interact in a complex dependent on RNA, which possibly serves as a bridge between them, and form abnormal cytoplasmic aggregates in cells [46]. Longer (CAG)_n_ tracts in *ATXN2* increase this interaction and, in conformity, as TDP-43 is a protein implicated in ALS, intermediate (CAG)_31–32_ *ATXN2* alleles increase the risk of ALS [46]. In ALS, ataxin-2 has shown an abnormal cytoplasmic accumulation instead of the normal diffuse pattern seen in unaffected spinal cord motor neurons, supporting the hypothesis that ataxin-2 is involved in ALS pathogenesis [46]. 

Interestingly, the *ATXN2* (CAG)_n_ is bidirectionally transcribed. Li and colleagues have identified an antisense (CUG)_n_ (*ATXN2-AS*) transcript in brain tissue of both unaffected and SCA2 affected subjects [10] (Figure 2). Notably, the (CUG)_exp_ *ATXN2-AS* transcript leads to cell death of primary mouse cortical neurons and forms RNA foci in SCA2 human cerebella and BAC-transgenic mice, which likely colocalize with Muscleblind-Like Splicing Regulator 1 (MBNL1), as detected in neuroblastoma cells transfected with the expanded (CAG)_n_ [10]. Although the *ATXN2-AS* with a (CUG)_>37_ is expressed in SCA2 human cerebella, both normal and expanded *ATXN2-AS* RNAs do not seem to be translated by RAN [10]. Furthermore, *ATXN2-AS* transcripts with intermediate (CUG)_31–32_ have been identified in ALS lymphoblastoid cell lines, suggesting an involvement of *ATXN2-AS* in ALS [10]. However, the role of *ATXN2-AS* in SCA2 and ALS is an important issue that needs further investigation.

### 2.2. ATXN7 Sense and Antisense Repeat Expression in SCA7

SCA7 is caused by a (CAG)_n_ expansion in exon 3 of the *ATXN7* [47,48]. Individuals affected with SCA7 carry one allele ranging from 37 to 400 CAG units with the larger sizes responsible for childhood disease onset [48] (Figure 1). In addition to cerebellar atrophy, SCA7 is also characterized by retinal degeneration [49]. Several transgenic mice expressing (CAG)_>37_ mirror the progressive ataxia and the cone-rod dystrophy seen in SCA7 individuals [50,51,52,53,54]. Ataxin-7 plays a role on the assembly and maintenance of transcriptional co-activation complexes TATA-binding protein-free TAFII/SPT3-TAF9-GCN5 acetyltransferase (TFTC/STAGA), which regulate the initiation of transcription [55,56,57]. Regarding retinal function, the polyQ region of ataxin-7 protein interacts with the transcription factor cone-rod homeobox protein (CRX), recruiting TFTC/STAGA complexes to the promoter region of photoreceptor-specific genes, regulating their transcription [51,58]. The expanded polyQ tract in ataxin-7 impairs TFTC/STAGA and CXR activities, originating transcriptional dysregulation and retinal dysfunction in transgenic mice expressing the mutant human full-length *ATNX7* cDNA in rod-photoreceptors, Purkinje cells and other neurons [50,51,53,57,58]. The expanded ataxin-7 polyQ tract also interacts with other proteins like the transcriptional coactivator CAMP-response element-binding protein (CBP), forming nuclear inclusions observed in Purkinje and retinal cells in SCA7 transgenic and knockin mice, contributing to retinal dysfunction [50,51,52,53,54,59,60] (Table 1). In concordance with ataxin-7 function, zebrafish *atxn7* translation inhibition also causes an early developmental impairment, leading to abnormal differentiation of photoreceptors and cerebellar neurons [61].

In contrast with transgenic mice expressing the human full-length *ATXN7* coding region in neurons, transgenic mice containing the repeat flanked by its endogenous regulatory regions showed that repeat instability is related to *cis*-regulatory elements in the *ATXN7* locus [62]. In particular, two functional CTCF-binding sites flank the (CAG)_n_ and are involved in SCA7 instability [62]. In this region, Sopher and colleagues have also discovered a human *ATXN7* alternative promoter and the spinocerebellar ataxia 7 antisense noncoding transcript 1 (*SCAANT1*) [11]. Notably, the *SCAANT1* is responsible for *ATXN7* transcriptional regulation. The authors found that, in normal size alleles, the CTCF-binding activates *SCAANT1* transcription, yielding convergent transcription with *ATXN7*, and consequently limiting *ATXN7* expression due to epigenetic modifications [11]. This transcriptional regulation mechanism is dysregulated in expanded (CAG)_n_ alleles. The repeat expansion leads to a decrease in CTCF-binding and, consequently, a decrease in *SCAANT1* transcription, which derepresses *ATXN7* [11] (Figure 2). The effect in human SCA7 cerebella that different repeat sizes play in this complex regulation has not been further studied. Intriguingly, the discovery of a noncoding antisense RNA repressing the transcription of an important protein involved in global transcriptional activity highlights the importance of antisense repeat RNAs as essential regulators of crucial mechanisms like transcription and the relevance of these targets for therapeutic intervention.

### 2.3. Bidirectional Transcription across the Repeat Region in SCA8

SCA8 is caused by a combined polymorphic CTA with a (CTG)_n_ expansion above 80 units in the 3′UTR of the *Ataxin-8 opposite strand* (*ATXN8OS*) [9,63,64,65,66] (Figure 1). *Drosophila* transgenic lines expressing normal and expanded (CUG)_n_ RNAs in photoreceptor neurons showed retinal neurodegeneration, strongly suggesting that the overexpression of RNA containing CUG repeats is sufficient to drive neuronal degeneration [67]. To further understand the molecular pathogenic mechanism in SCA8, Moseley and colleagues have generated BAC-transgenic mice expressing the human full-length *ATXN8OS* with (CTG)_104_ under its endogenous promoter, recapitulating the motor dysfunction and cerebellar deficits seen in SCA8 [9]. Remarkably, these authors found that the CTG/CAG repeat expansion is bidirectionally transcribed in SCA8 BAC-transgenic mice, forming polyQ ubiquitin-positive intranuclear inclusions resulting from (CAG)_n_ translation, which was also verified in human SCA8 brain tissue [9] (Figure 2). From the *ATXN8OS*, the expanded (CTG)_n_ is transcribed in a noncoding transcript, suggesting that both protein and RNA gain-of-function mechanisms contribute to SCA8 [9]. The expanded (CUG)_n_ forms ribonuclear inclusions that colocalize with MBNL1 in cerebella of SCA8 subjects and BAC-transgenic mice, leading to misplicing [68] (Table 1). Moreover, (CAG)_n_ expanded transcripts are RAN-translated generating homopolymeric peptides of polyA and polyserine (polyS) (Table 1). Therefore, in addition to polyQ, polyA and polyS peptides also form inclusions in cerebella of SCA8 human brains and BAC-transgenic mice, further supporting a peptide gain-of-function in SCA8 [5,69].

In the human genome, the SCA8 (CAG)_n_ is adjacent to the promoter of actin-binding protein gene *Kelch-like 1* (*KLHL1*) [70]. This gene has an antisense RNA (*KLHL1AS*) that holds the *ATXN8OS* (CUG)_n_ at the 3′end. Interestingly, it has been suggested that *KLHL1AS* regulates *KLHL1* expression in the cerebellum, raising the hypothesis that the function of *KLHL1AS* and *KLHL1* could be dysregulated in SCA8 [70,71]. In fact, a mouse with a deletion of both *Klhl1* gene and *Klhl1as* promoter showed gait abnormalities, motor incoordination and dendritic atrophy, supporting the hypothesis that *KLHL1* and/or its antisense have a role in SCA8 [72]. Although the phenotype observed in these mice suggests a *KLHL1* loss-of-function in SCA8, the downregulation of human *KLHL1* needs to be further investigated in human tissues of SCA8 subjects [73]. Moreover, the human *KLHL1AS* RNA harbors the (CUG)_n_, but the mouse *Klhl1as* RNA ends upstream the (CUG)_n_ [71]. Therefore, this knockout mouse might not be the most suitable model to study the involvement of *KLHL1AS* on *KLHL1* expression and the role of the (CTG)_n_ in this regulation [73]. Altogether, both RNA and protein-mediated mechanisms are seemingly involved in SCA8, but how each of these mechanisms contributes to the disease phenotype is not clear. Further investigation of the sequestered proteins in cerebellar nuclear inclusions and RNA foci could give insight into the cellular pathways impaired in this disease. 

### 2.4. Pentanucleotide Repeats in Introns of Genes Transcribed from Opposite Strands in SCA31

In 2009, Sato and colleagues found a (TGGAA)_n_ insertion causing SCA31 [12] (Figure 1). This insertion is in a shared intronic region between *Brain expressed associated with NEDD4-1* (*BEAN1*) and *Thymidine kinase 2* (*TK2*) genes, which are transcribed from opposite DNA strands [12]. In *BEAN1*-orientation, the transcribed (TGGAA)_n_ insertion forms nuclear RNA aggregates in Purkinje cells from SCA31 individuals [12,74]. Consistent with the SCA31 phenotype, *Drosophila* lines expressing (TGGAA)_80–100_ in the nervous system showed an early lethality and progressive motor abnormalities [75], suggesting that the (TGGAA)_n_ insertion could impact neuronal development. In the *Drosophila* eye, (TGGAA)_80–100_ expression led to eye degeneration, accumulation of nuclear (UGGAA)_n_ aggregates and poly-tryptophan-asparagine-glycine-methionine-glutamic acid (polyWNGME) pentapeptide repeat proteins (PPR) in imaginal discs [75]. These abnormal PPRs have also been detected in cell bodies and dendrites of SCA31 Purkinje cells [75]. The (UGGAA)_n_ insertion binds in vitro to serine/arginine-rich splicing factors (SRSFs) 1 and 9, and in vivo to *C9ORF72* FTD/ALS-related proteins such as Fused in sarcoma (FUS), heterogeneous nuclear ribonucleoproteins A2/B1 (hnRNP A2/B1) and TDP-43 [12,75,76] (Table 1). Remarkably, concomitant expression of (UGGAA)_n_ and TDP-43 protein rescued eye degeneration in transgenic flies by decreasing RNA foci formation and PPR translation, suggesting that TDP-43 could function as an RNA chaperone [75,76]. Similar studies have been performed with FUS and hnRNP A2/B1 proteins and the results are identical to those obtained with TDP-43, indicating that these proteins could also function as RNA chaperones [75]. In the opposite orientation, the (TGGAA)_n_ is transcribed within an intron of *TK2* gene and though no RNA foci or PPR proteins have been reported, rescue of SCA31 phenotype could require targeting both sense and antisense repeats.

## 3. *FXN* Antisense Transcript in Friedreich Ataxia

Friedreich ataxia (FRDA), an autosomal-recessive ataxia with a high prevalence worldwide, is mainly caused by a biallelic (GAA)_n_ expansion in intron 1 of the *frataxin* (*FXN*) gene [24]. FRDA alleles have over 66 GAA repeat units, leading to sensory neuron loss, progressive motor and cognitive deficits, diabetes and hypertrophic cardiomyopathy that culminate in an early death [77,78,79] (Figure 1). Expansion of the (GAA)_n_ causes a decrease of *FXN* mRNA and protein through histone modifications, possibly associated with heterochromatin formation in the repeat flanking region, in human FRDA brain tissue [80,81,82,83]. In FRDA lymphoblasts, the repressive chromatin, with increased tri-methylation at the 27th lysine residue of histone 3 (H3K27me3) and decreased acetylation at the 5th lysine residue of histone 4 (H4K5Ac) in the *FXN* promoter and GAA-upstream regions, agrees with the *FXN* transcriptional repression and FXN protein deficiency [84]. Frataxin is a crucial protein for mitochondrial iron metabolism and its deficiency leads to oxidative stress and mitochondrial dysfunction [85]. Remarkably, transcription factors, as serum response factor (SRF) and transcription factor AP2 (TFAP2), with binding sites in exon 1 together with regulatory elements in intron 1 regulate *FXN* transcription. In FRDA lymphoblasts, overexpression of SRF and TFAP2 leads to an increase of *FXN* transcription [86], suggesting that the (GAA)_n_ expansion could cause alterations in the binding of these transcription factors. 

A CTCF-binding site in the *FXN* promoter region has shown an enrichment of CTCFs in fibroblast cell lines from unaffected individuals, contrarily to FRDA fibroblast cells, which correlates with heterochromatin formation [87]. Spanning this CTCF-binding site, there is an antisense transcript named as *FXN Antisense Transcript-1* (*FAST-1*), whose expression is 2-fold increased, in FRDA fibroblast cells [87]. Intriguingly, siRNA-mediated knockdown of CTCF protein, in control fibroblasts, led to a decrease of *FXN* transcription concomitant with an increase of *FAST-1* mRNA, recapitulating the *FXN* and *FAST-1* mRNA levels detected in FRDA fibroblasts [87]. Accordingly, overexpression of *FAST-1*, in HeLa transfected cells, led to *FXN* mRNA and protein decrease, CTCF-binding depletion and histone methylation enrichment in the *FXN* promoter region [19]. In FRDA fibroblasts, shRNA-mediated knockdown of the *FAST-1* mRNA originated increased *FXN* transcription and mitochondrial aconitase activity, restoring frataxin function [19]. The pathogenic mechanism in FRDA is not completely understood, but the *FAST-1* transcript, which does not span the repeat expansion, seems to influence this mechanism. *FAST-1* could play a role in *FXN* transcription regulation, as occurs in SCA7, and be a successful target for therapeutic intervention.

## 4. Antisense Expression in Hexanucleotide Repeat Expansion Diseases *C9ORF72* FTD/ALS and SCA36

*C9ORF72* FTD/ALS and SCA36 are autosomal-dominant neurodegenerative diseases, characterized by dementia and motor neuron degeneration or ataxia, respectively. Although these diseases present differences in clinical presentation, both are caused by similar noncoding hexanucleotide repeat expansions. A (GGGGCC)_n_ expansion, of >30 repeat units, in intron 1 of chromosome 9 open reading frame 72 (*C9ORF72*) causes *C9ORF72* FTD/ALS, whereas SCA36 is caused by a (TGGGCC)_n_ expansion of 650 or more repeats in intron 1 of the nucleolar protein 56 (*NOP56)* [88,89,90,91] (Figure 1).

The transcription of *C9ORF72* and *NOP56* hexanucleotide repeats initiates a cascade of parallel pathogenic mechanisms that include the sequestration of essential neuronal RBPs in RNA foci and the production of toxic dipeptide repeat proteins (DPRs) by RAN translation [13,16,92,93] (Figure 2 and Table 1). In addition to these RNA-mediated toxic mechanisms, the decreased levels of *C9ORF72* transcripts in brain tissue of *C9ORF72* FTD/ALS individuals, together with the characteristic neuropathology observed in animal models for gene loss-of-function, indicate that also *C9ORF72* haploinsufficiency is implicated in this disease [94,95]. In SCA36, the cerebellum of affected subjects shows an increase in *NOP56* transcript and protein discarding gene haploinsufficiency [14]. 

The mechanism of RNA-mediated toxicity implicated in *C9ORF72* FTD/ALS and SCA36 is not triggered only by transcription of expanded hexanucleotide repeats in *C9ORF72* and *NOP56* gene expression context, but also by antisense transcripts [13,14,96,97]. The *C9ORF72* has five transcript start sites (TSSs) in sense orientation and three in antisense orientation [96,98]. The (GGGGCC)_n_ is transcribed from two *C9ORF72* TSSs, whereas the antisense (GGCCCC)_n_ is transcribed from one TSS [96]. Notably, BAC-transgenic mice expressing mosaics of 100–1000 GGGGCCs within the human *C9ORF72* gene and containing gene flanking sequences have presented both sense and antisense RNA foci and RAN DPRs with a brain distribution similar to *C9ORF72* expansion carriers, but no detectable neurodegeneration or behavioral abnormalities [99]. Remarkably, another *C9ORF72* BAC-transgenic mouse containing ~500 repeats, but without 3′ gene sequences have also shown RNA foci and RAN DPRs, but again no neurodegeneration or behavioral changes [100]. On the other hand, BAC-transgenic mice with transgenes of 500 or both 500 and 32 repeats, within the human full-length *C9ORF72* flanked by regulatory regions, driving sense and antisense expression using the endogenous human promoters, presented neurodegeneration and motor dysfunction. In this mouse model, antisense expression was upregulated in frontal cortex, as seen in autopsy tissue from *C9ORF72* expansion carriers, indicating that the expansion regulates antisense expression [101]. Notably, in this model, in contrast to sense RNA foci that are found throughout the brain, antisense RNA foci accumulate in vulnerable *C9ORF72* FTD/ALS cell populations like the upper motor neurons of the motor cortex. This BAC mouse model shows decreased survival, paralysis, muscle denervation, motor neuron loss, anxiety-like behavior, and cortical and hippocampal neurodegeneration. What seems to distinguish these latter BAC-transgenic mice from the previous mice, leading to the manifestation of the expected neurological phenotype, is the large region flanking both sense and antisense promoters that could facilitate interaction of regulatory regions with promoters, originating robust expression of transcripts. The relevance of antisense transcripts in the severity of the disease is supported by the milder phenotype when only overexpression of sense (GGGGCC)_66_ throughout the central nervous system is achieved by means of somatic brain transgenesis mediated by adeno-associated virus [102].

The *NOP56* transcriptional landscape has not been fully investigated, however, in addition to nuclear RNA foci triggered by the sense (TGGGCC)_n_ expansion in brain tissue of SCA36 affected individuals, RNA foci containing the antisense expansion have been detected in brains of transgenic mice for the (TGGGCC)_n_ expansion, suggesting that antisense RNA may have a role in SCA36 pathology [13,103].

Interestingly, it has been suggested a modest role for *C9ORF72* hexanucleotide repeat RNA in the neurodegenerative phenotype [104]. A *Drosophila* model expressing ~800 GGGGCCs, interrupted with stop codons to avoid RAN translation, showed that the expanded repeat RNA sequesters RBPs, though it is not toxic in this organism. Similarly, an antisense RNA with ~100 CCCCGG repeats alone does not trigger a neurodegenerative phenotype [104].

The transcription of both sense and antisense expanded repeats leads to translation in RAN DPRs in neuronal brain tissue of *C9ORF72* FTD/ALS individuals and BAC-transgenic mice [96,97,99,100,101]. This RAN translation produces five DPRs, polyGA (glycine-alanine) and polyGR (glycine-arginine) from sense, polyPR (proline-arginine) and polyPA (proline-alanine) from antisense and polyGP (glycine-proline) from both sense and antisense transcripts [16,96,97]. All these DPR species are widely found in cytoplasmic neuronal inclusions in several brain regions, including the frontal cortex, of *C9ORF72* FTD/ALS subjects [16,96]. In *C9ORF72* antisense orientation, the translation in polyPR and polyGP may occur from one and three putative ATG-start codons, respectively, however, in vitro studies using cell lines transfected with a plasmid containing a (GGCCCC)_n_ expansion have confirmed that RAN translation from the antisense strand drives polyPR and polyGP production [96,97]. 

The similarities between *C9ORF72* FTD/ALS and SCA36 go beyond neurodegeneration. The hexanucleotide repeats share five nucleotides by each repeat unit, which has set the stage for the investigation of DPRs resulting from RAN translation in SCA36 [13,14]. Remarkably, polyGP and polyPR are produced in *C9ORF72* FTD/ALS and SCA36 brain tissue and both are encoded from antisense repeat strand [13,14] (Table 1). Furthermore, studies in *Drosophila* and zebrafish models using codons, other than the *C9ORF72* hexanucleotide repeat, to encode the DPRs showed that the arginine-rich dipeptide species, including polyPR, are highly toxic in these organisms [105,106]. In fact, the toxic polyPR peptides form aggregates in the cytoplasm of cerebellar granule cells of SCA36 and *C9ORF72* FTD/ALS subjects [13,14]. The polyGP is more abundant in cells and neuronal tissues of SCA36 than in *C9ORF72* FTD/ALS subjects, however, contrarily to polyGP insoluble cytoplasmic aggregates found in *C9ORF72* FTD/ALS, they remain soluble in the cytoplasm of neuronal cells in SCA36 [13,14].

Although several studies have given insight on how the hexanucleotide repeat expansion in *C9ORF72* and *NOP56* antisense strands could contribute to the neurodegenerative phenotype, the biological function of the antisense transcripts encompassing these repeats remains poorly understood. Interestingly, in *C9ORF72* FTD/ALS, a decrease in *C9ORF72* expression in brain and monocytes of affected individuals is accompanied by an increase in antisense transcript expression, suggesting that antisense transcripts could be involved in *C9ORF72* transcription regulation [96,98]. The findings described above indicate that successful therapies for these two diseases have to take into account antisense repeat transcripts.

## 5. Antisense Expression in Neuronal CGG Repeat Diseases 

### 5.1. Antisense Transcript Spanning FMR1 Repeat Region in FXTAS 

FXS and FXTAS result from a (CGG)_n_ expansion in the 5′UTR of the *FMRP translational regulator 1* (*FMR1*) gene [107,108]. FXS subjects carry a full-mutation allele with >200 CGGs causing developmental delay and intellectual disability, often accompanied by autism spectrum disorder, whereas FXTAS-associated alleles harbor a premutation ranging from 55 to 200 CGG repeat units, developing late-onset progressive ataxia, tremor and cognitive impairment [107,108] (Figure 1). In FXS, the (CGG)_>200_ expansion spanning the promoter region triggers its hypermethylation, leading to *FMR1* gene silencing [109,110,111,112]. In contrast to *FMR1* loss-of-function in FXS, a slight increase of *FMR1* mRNA levels has been detected in FXTAS lymphoblastoid cells, supporting an RNA gain-of-function leading to the disease [113]. However, the repeat premutation size leads to a decrease in translation efficiency of the *FMR1* mRNA and, therefore, identical levels of FMR1 protein (FMRP) have been detected in FXTAS lymphoblastoid cells compared with controls [114,115]. Several studies have shown that *FMR1* mRNA forms ubiquitin-positive intranuclear inclusions in neuronal and glial cells in FXTAS brain tissues [116,117,118] and (CGG)_98_-knockin mice [118,119,120], suggesting a neurotoxicity driven by RNA. The (CGG)_n_ premutation expressed in Purkinje cells leads to neurodegeneration in mice, independent from *FMR1* gene context [121], demonstrating that the premutation RNA is a key player in this disease. In fact, a *Drosophila* model expressing a human 5′UTR fragment with (CGG)_90_ evidenced FMRP reduction, presenting ubiquitin-positive intranuclear inclusions in photoreceptor neurons and progressive neurodegeneration [122]. In this fly model and in FXTAS human cortical tissues, RBPs involved in RNA processing like the hnRNP A2/B1, Purine-rich binding protein-α (Pur α), MBNL1 and CUGBP Elav-Like Family Member 1 (CELF1) are sequestered by the premutation mRNA in intranuclear inclusions, preventing them from performing their normal functions [118,123,124,125]. The premutation *FMR1* RNA is also RAN-translated into polyA and polyglycine (polyG) peptides, which originate ubiquitin-positive intranuclear inclusions colocalizing with p62 that could also contribute to neurodegeneration in *Drosophila* and FXTAS brain tissue [126,127,128].

In FXTAS, in addition to (CGG)_55–200_ RNA and protein-mediated toxicity, Ladd and colleagues discovered two antisense promoters flanking the (CCG)_n_ and one antisense transcript (*ASFMR1*) spanning the repeat [15] (Figure 2). Similar to *FMR1* mRNA, the *ASFMR1* RNA levels are increased mainly in FXTAS autopsy brain tissue, endorsing a role for *ASFMR1* in FXTAS [15]. Moreover, a *Drosophila* model expressing a (CCG)_90_ has shown retinal neurodegeneration, suggesting a toxic role for the *ASFMR1* premutation [129]. In agreement, the spliced and polyadenylated *ASFMR1* RNA with the (CCG)_n_ premutation is transported to the cytoplasm and translated into a polyproline (polyP) peptide from an AUG-start codon [126,130]. PolyP and polyA peptides are RAN-translated, forming ubiquitin-positive neuronal inclusions in FXTAS brain tissue [127,130]. Like in SCA7 [11], there are CTCF-binding sites flanking the CGG/CCG repeat [15], but their role in *FMR1* mRNA upregulation or silencing is poorly understood. Interestingly, the co-expression of expanded (CCG)_n_ and (GGC)_n_ in *Drosophila* decreased their RNA levels through Dicer and Argonaute-2, reverting the neurodegenerative phenotype [129], providing clues for a future therapeutic strategy for FXTAS.

### 5.2. NOTCH2NLC Repeat Expansions in NIID and Expanded Noncoding RNA LOC642361 in OPML1

Novel noncoding CGG repeat expansions have recently been discovered causing NIID and OPML1, two neurodegenerative diseases that share with FXTAS the symptoms of ataxia and tremor, and the presence of ubiquitin-positive neuronal inclusions [20,21]. Broadly, muscle weakness, dementia and parkisonism are common clinical signs seen in NIID subjects [20,131], whereas OPML1 is characterized by oculopharyngeal myopathy, limb weakness and leukoencephalopathy [21]. In NIID, there is an expanded (CGG)_n_ larger than 66 units in the 5′UTR of *Notch 2 N-terminal like C* (*NOTCH2NLC*), leading to sporadic and familial NIID [20] (Figure 1). The *NOTCH2NLC* gene, highly expressed in the brain, is thought to play a role in neuronal proliferation and differentiation [20,132,133]. Interestingly, antisense transcripts have been detected spanning the (CGG)_n_ region, in fibroblasts of NIID individuals [134]. Surprisingly, these antisense transcripts have not been found in fibroblasts from unaffected subjects [134], suggesting that bidirectional transcription occurs only in expanded alleles. DNA methylation changes in the expanded (CGG)_n_ allele could lead to this antisense transcription, however, the authors did not find significant differences in CpG methylation profiles between leukocytes and fibroblasts from NIID subjects and controls [20,134]. On the other hand, another study has shown a tendency for DNA hypermethylation of this repeat region in NIID brain tissue [21]. The size of the (CGG)_n_ could account for the differences in DNA methylation seen in these two studies. Although no *NOTCH2NLC* expression changes have been detected, several genes with neuronal functions have shown differences in fibroblasts [134], suggesting that the expanded (CGG)_n_ could dysregulate neuronal gene expression in NIID.

The first (CGG)_n_ expansion in a long noncoding RNA bidirectionally transcribed has recently been found in *LOC642361/NUTM2B-AS1* causing OPML1 [21] (Figure 1). It remains to be seen if both sense and antisense RNAs contribute to disease by toxic RNA and RAN-peptides.

## 6. Antisense Repeat Expression in HD and HDL2

HD and HDL2 are two clinically and pathologically similar neurodegenerative diseases, characterized by movement, cognitive and psychiatric disturbances [135]. The individuals affected by these diseases present a degeneration of the cerebral cortex and striatum, endorsing a common pathogenic mechanism in both pathologies [135]. HD is caused by an expanded (CAG)_>36_ in exon 1 of *huntingtin* (*HTT*) gene [136], whereas HDL2 is caused by a noncoding (CTG)_>40_ in exon 2A of *junctophilin-3* (*JPH3*) gene [137,138] (Figure 1). HD mouse and cell models have demonstrated that truncated N-terminal HTT fragments with expanded polyQ form intranuclear ubiquitin-positive inclusions in neurons and dystrophic neurites, as observed in HD brain tissues [139,140,141,142,143,144]. These mouse models also recapitulate the typical progressive loss of balance and motor impairment, tremor and involuntary movements present in HD subjects [139,140,141]. Although the function of HTT protein is not well established [145], the expanded HTT forms nuclear aggregates in neurons of HD transgenic mouse models, contrarily to the characteristic diffuse pattern of normal HTT protein in the cytoplasm, being this mislocalization involved in HD pathogenesis [140,141,142]. Concomitantly, several studies have revealed that expanded HTT intranuclear inclusions colocalize with many cellular proteins with a role in transcription such as CBP, histone deacetylase complex subunit Sin3a (mSIN3a) and TATA-binding protein (TBP), in HD human cortex tissues [125,146,147,148,149,150] (Table 1). Furthermore, the *HTT* mRNA with the expanded (CAG)_n_ gains the ability to sequester RBPs, such as MBNL1 in HD fibroblasts and nucleolin in HD transgenic mouse brains, possibly causing a splicing misregulation of MBNL1 target pre-mRNAs, nucleocytoplasmic transport impairment and nucleolar stress [151,152,153,154].

In 2011, Chung and colleagues discovered the existence of a natural antisense transcript spanning the (CTG)_n_ across the *HD* locus (*HTTAS1*) [17]. The authors have detected a decrease in *HTTAS1* transcripts and identical *HTT* RNA levels in HD brain tissues [17]. Unexpectedly, another report has shown that HTT protein levels are identical between *post-mortem* brain tissues from subjects with an adult HD onset and controls, but HTT protein is reduced in brain protein extracts of juvenile onset individuals [155]. Therefore, juvenile HD onset is characterized by larger repeat expansions that seem to influence the expression of HTT protein and potentially *HTTAS1* transcript. Chung and colleagues have also shown that *HTTAS1* is able to regulate *HTT* RNA degradation on a RISC-dependent manner [17] (Figure 2). Moreover, two predicted CTCF-binding sites and two Sp1 and Ap2 transcription factor-binding sites have been reported in the 5′region of *HTTAS1* promoter [17], raising the hypothesis of a transcriptional regulation in HD similar to *ATXN7*.

HD has been one of the first diseases for which RAN-peptides have been investigated [5]. Both sense (polyA and polyS) and antisense (polycysteine (polyC) and polyleucine (polyL)) RAN-peptides accumulate into cytoplasmic, perinuclear and nuclear aggregates in the cerebellum and cortex, and mainly in nuclear aggregates in cerebral and cerebellar white matter of HD human tissue, likely contributing to the disease [156].

In HD mice expressing exon 1 of *HTT* under its endogenous promoter and in HD human autopsy tissue, small RNAs containing CAG repeats (sCAGs) have been identified [157]. In fact, expanded *HTT* mRNA forms hairpins that are cleaved by a Dicer-dependent mechanism and, consequently, the resultant neurotoxic sCAGs of approximately 21 nucleotides could silence other RNAs containing CUG repeats, disrupting their functions [157,158]. Independently, another study has also shown that Dicer-processed CAG/CUG repeat RNAs could interfere with the stability of other gene transcripts, leading to neurodegeneration in *Drosophila* [159].

Due to clinical and neuropathological similarities between HD and HDL2, the same neurodegenerative mechanisms could underlie both diseases. JPH3 protein is highly expressed in Purkinje cells and is involved in the formation of junctional membranes, therefore, the loss of JPH3 could partially explain motor dysfunction in HDL2 [18,160,161,162]. Although no evident morphologic and functional abnormalities have been detected in cerebellar neurons of *Jph3* knockout mice, these animals have shown motor dysfunction, supporting a role for reduced JPH3 protein in HDL2 [18,162]. 

In brain tissue of HDL2 subjects, expanded *JPH3* transcripts form RNA aggregates with MBNL1, with consequent misplicing of MBNL1 mRNA targets in frontal cortex [163] (Table 1). In concordance, HDL2 BAC-transgenic mice expressing the full *JPH3* locus with a (CTG)_116_ has also shown cortical (CUG)_n_ RNA foci colocalizing with Mbnl1 accompanied by cortex atrophy and motor impairment, mimicking the HDL2 phenotype [164]. Notably, these transgenic mice allowed the identification of a *HDL2* antisense CAG transcript translated into an aberrant polyQ expanded protein, forming ubiquitin-positive intranuclear inclusions in cortex, hippocampus and striatum of HDL2 BAC-transgenic mice [164]. The authors demonstrated also that these nuclear inclusions are able to sequester the transcriptional coactivator CBP in HDL2 human cortical neurons, suggesting a transcription dysregulation in this disease [164]. In this regard, Seixas and colleagues have shown that the antisense (CAG)_n_ is transcribed, however, the expanded (CAG)_n_ transcript and the polyQ protein were not detected in autopsy brain tissue from HDL2 individuals [18], probably due to the lower levels in human brain tissue compared with the transgenic mice. This clearly demonstrates bidirectional transcription of the *JPH3* repeat in unaffected chromosomes. Further investigation of a potential regulatory mechanism involving both *JPH3* sense and antisense expression, as occurs in HD, may contribute to explain how JPH3 protein expression is regulated (Figure 2).

Notably, the knowledge gathered indicates that antisense repeat transcripts *HTTAS1* and *HDL2AS* have the potential to be targets for efficient and successful therapies in HD and HDL2, respectively.

## 7. Antisense Repeat Expression in DM1 and DM2

DM1 and DM2 are two clinically similar neuromuscular diseases [165]. While a (CTG)_n_ expansion, ranging from 50 to several thousand CTGs, in the 3′UTR of *DMPK* causes DM1 [166,167,168], a (CCTG)_n_ expansion, from 75 to 11,000 repeats, in intron 1 of *CNBP* is responsible for DM2 [169] (Figure 1). Very large expansions of (CTG)_>1000_ cause a more severe type of disease, congenital DM1, characterized by pronounced motor and cognitive disability during development, which is associated with childhood onset and early death [170]. Clinical similarities between DM1 and DM2 individuals include cardiac dysfunction, cataracts formation, skeletal muscle abnormalities and neuronal involvement [165]. In DM1, several studies have shown that the (CUG)_>90_ RNA forms nuclear aggregates, causing *DMPK* mRNA and protein reduction, as seen in DM1 muscle [171,172,173]. Accordingly, an homozygous mouse model with *Dmpk* disruption has shown some DM1 clinical features, such as progressive weakness, abnormalities in skeletal muscle, skeletal myopathy and dysfunction in cardiac conduction [174,175]. Although this mouse presented some clinical features of DM1, the complex muscle phenotype observed in DM1 subjects was not fully recapitulated, indicating that *DMPK* haploinsufficiency is not sufficient to explain the DM1 phenotype [174,175]. On the other hand, a transgenic mouse expressing a *human skeletal actin* fragment with a 3′UTR (CTG)_>250_, mimicking the location of the repeat expansion in *DMPK*, in a muscle-specific gene, developed limb difficulties, myotonia and myopathy, indicating that the expanded (CUG)_n_ RNA is sufficient to cause DM1 clinical features in muscle [176]. However, the typical muscle atrophy and weakness observed in DM1 individuals were not detected in these animals [176]. 

The hypothesis of *CNBP* haploinsufficiency for DM2 has also been investigated by the generation of two *Cnbp* knockout mice. These animals presented an increased lethality associated with homozygous *Cnbp* deletion, which demonstrates that CNBP expression is crucial during development [177,178]. The first mice developed several DM2 characteristics such as myotonia, cataracts, cardiac arrhythmia and abnormalities in skeletal and heart muscle tissues [177], whereas the other presented muscle abnormalities with progressive cell loss in adult, muscle wasting and mislocalization of CNBP from the cytoplasm to the membrane [178]. In fact, reduction of CNBP expression in the cytoplasm accompanied by aberrant membrane location has been detected in DM2 myoblasts and muscle biopsy tissues [179,180]. In agreement with the pathogenic mechanisms found in DM1, the (CCUG)_>75_ RNA in DM2 also forms nuclear RNA foci in muscle of affected individuals [169]. Nuclear RNA aggregates colocalizing with MBNL proteins have been detected in muscle tissues from both DM1 and DM2 subjects [181]. Interestingly, nuclear RNA foci also colocalize with MBNL1 and MBNL2 proteins in cortical neurons [125] (Table 1). Both DM1 and DM2 phenotypes correlate with MBNL downregulation and CELF1 upregulation, which leads to splicing misregulation of MBNL and CELF1 target pre-mRNAs in neuronal and cardiac muscle cells [182,183,184,185,186,187,188]. MBNL loss-of-function or its sequestration by expanded (CUG)_n_ has also been observed in mouse [189], *Drosophila* [190] and zebrafish [191] models. Remarkably, MBNL1 loss-of-function is an important mechanism involved in the clinical phenotype of these diseases. A knockout mouse carrying *Mbnl1* gene with a deletion in the CUG repeat-binding site presented misplicing of several key mRNAs, such as that encoding chloride channel and cardiac troponin T, recapitulating several DM1 and DM2 features in the muscle and eye [189]. Furthermore, *Drosophila* expressing (CUG)_480_ in the eye and muscle also mimicked the degeneration of these tissues and presented nuclear RNA foci colocalizing with MBNL proteins [190]. Intriguingly, the MBNL1 overexpression in this fly model rescued the degenerative phenotype in eye and muscle [190], hinting a possible therapy for these diseases. Zebrafish embryos injected with the *3′DMPK* RNA holding a (CUG)_91_ also formed nuclear RNA foci, developed morphological and behavioral abnormalities and showed transcriptional dysregulation during early development [191]. Supporting an RNA-mediated mechanism, several RBPs involved in transcription and RNA metabolism colocalize with nuclear RNA foci in DM1 cortical neurons and in DM1 and DM2 human-derived cells [125,192] (Table 1).

Bidirectional transcription of a repeat expansion has first been discovered by Cho and colleagues, for DM1. These authors demonstrated that the antisense transcript spanning the repeat is converted into siRNAs, able to recruit DNA and histone methyltransferases, in particular the heterochromatin protein 1γ (HP1γ), leading to heterochromatin formation [8]. Abnormal heterochromatin formation had previously been seen in primary DM1 myoblasts and fibroblasts, dysregulating transcriptional activity of *DMPK* and the neighboring *SIX5* gene [170,193,194,195,196]. Unexpectedly, there is an increase of *DMPK* expression in cells of congenital DM1 individuals that present increased CpG methylation of CTCF-binding sites flanking the (CTG)_n_ substantially reducing binding of CTCFs [197]. Similar to *ATXN7*, Nakamori and colleagues discovered a *DMPK* alternative promoter upstream the (CTG)_n_, that is regulated by an antisense CAG repeat transcript [198]. These authors found that in muscle of congenital DM1, reduction in CTCF-binding suppresses antisense CAG transcription and promotes alternative *DMPK* transcription, leading to increase in (CUG)_n_ RNA and enhancing RNA toxicity (Figure 2). Interestingly, transgenic mice generated by Huguet and collaborators showed that both (CUG)_n_ and (CAG)_n_ DM1 transcripts form distinct ribonuclear foci, both potentially contributing to the disease [199]. Moreover, the antisense CAG siRNAs are able to reduce the sense (CUG)_n_ toxicity, decreasing the (CUG)_n_ RNA foci formation and splicing abnormalities [198]. Altogether, the authors proposed that the hypermethylation of CTCF-binding sites leads to *DM1* locus transcriptional dysregulation with a consequent decrease of antisense CAG siRNAs and increase of expanded (CUG)_n_-mediated toxicity, whose downstream effects lead to muscle deficiencies in congenital DM1 [198] (Figure 2).

In DM1, the expanded CAG transcript is RAN-translated into polyQ peptides in cardiac myocytes of DM1 mice [5] (Table 1). More recently, it has also been demonstrated that antisense transcription occurs across *DM2* locus and the antisense transcripts containing the expanded (CAGG)_n_ are upregulated in DM2 human brain tissue [6]. Both sense (CCUG)_n_ and antisense (CAGG)_n_ expanded RNAs are RAN-translated producing toxic poly-leucine-proline-alanine-cysteine (polyLPAC) and poly-glutamine-alanine-glycine-arginine (polyQAGR) tetrapeptides, respectively, able to induce cell death in human transfected cells (Table 1)**.** In DM2 tissue, polyLPAC aggregates have been mainly detected in the cytoplasm of neurons and glia, and polyQAGR in oligodendrocyte nuclei, potentially contributing to the disease. Interestingly, the antisense (CAGG)_n_ also binds to hnRNP A1, an RBP implicated in RNA processing [6]. 

Taken together, this emerging evidence in DM1 and DM2 strongly indicates that antisense repeat RNAs are key players in several molecular pathways that when dysregulated cause disease, being important targets for the development of therapeutic strategies.

## 8. Therapeutic Strategies Targeting Toxic RNAs 

A potential therapeutic strategy to target mutant repeat RNAs is based on RNA interference (RNAi) molecules that are complementary RNAs to the mutant transcripts, leading to the formation of double-stranded RNAs and, consequently, to their RISC-mediated degradation or translation repression. These molecules can be delivered directly into the cell as miRNAs or siRNAs, or indirectly as short hairpin RNAs (shRNAs) that are delivered generally by adeno-associated viruses (AAV) [200]. Several RNAi molecules have been tested in animal and cellular models for SCA7 [201,202,203], HD [204,205,206,207] and DM1 [208,209,210], reducing the toxicity of the mutant repeat RNAs. Remarkably, a miRNA targeting the *HTT* mRNA delivered using an AAV5 has been tested in the striatum of HD rodents and minipig models, leading to mutant mRNA and protein reduction, with consequent reduction of abnormal protein aggregation, thereby ameliorating the HD phenotype [204,205]. This AAV5-miHTT is currently a promising therapy for HD, with clinical phase I/II studies currently ongoing [211,212]. The delivery of RNAi molecules is facilitated by the use of viral vectors, which can be considered as an advantage since a single dose may be sufficient for treatment. 

Other promising therapies to target mutant repeat RNAs are based on antisense oligonucleotides (ASOs) [213]. These ASOs, composed by a DNA sequence flanked by RNA nucleotides, are complementary to the target RNA, leading to its degradation, in the nucleus or cytoplasm by RNase H [214]. The challenges in these treatments are ASOs stability and delivery to muscle and brain. Therefore, several chemical modifications and delivery carriers may improve stability and uptake of these molecules [215,216]. In recent years, ASOs targeting sense RNAs have successfully been tested on mouse and human cellular models, showing a decrease of toxic transcripts and a partial rescue of the disease phenotype, as in SCA2 [217,218], SCA7 [219], HD [220], DM1 [221] and *C9ORF72* FTD/ALS [222,223,224,225]. ASOs have shown their potential as a therapeutic strategy in preclinical studies for SCA2 [217,218], and in clinical studies for DM1, HD [220] and *C9ORF72* FTD/ALS. In SCA2, ASOs decrease the toxic RNA and protein, improving the motor function in mice [217]. Moreover, three different ASOs have been studied in clinical trials for HD; one of them, in phase III, consists in a nonallele-specific ASO targeting both wild-type and mutant *HTT* RNAs, in which no pathogenic effect caused by wild-type HTT protein deficiency was detected [220], and the remaining are in phase I/II and they specifically target mutant *HTT*, using single nucleotide polymorphisms associated with the disease haplotype [226]. In DM1, an ASO targeting the mutant *DMPK* RNA is currently in phase I/II clinical trial, due to the observed successful rescue of muscle dysfunction in mouse models [221]. More recently, a potential ASO for *C9ORF72* FTD/ALS [222], which reduces RNA foci formation and dipeptide inclusions in *C9ORF72* FTD/ALS transgenic mice, has been in clinical phase I studies. The presented therapeutic strategies are based on ASOs that specifically target the toxic sense transcripts. However, in several diseases, including HD, SCA2, DM1 and *C9ORF72* FTD/ALS, both sense and antisense repeats contribute with toxic species, thus targeting both mutant sense and antisense RNAs could be crucial to improving the success of therapies designed to revert the disease phenotype and/or delay disease progression. 

## 9. Conclusions and Future Perspectives

Antisense repeat RNAs have been identified in several genes associated with neurodegenerative and neuromuscular diseases caused by repeat expansions. Although enormous efforts have been made to understand the complex mechanisms underlying these diseases, the contribution of mutant antisense repeat RNAs remains elusive. This is due to challenges regarding the assessment of their expression levels that tend to be low, the recognition of their cell and tissue-specific expression that probably varies from development to aging hindering the discovery of their biological function and role in disease. In addition, the scarcity of animal models with bidirectional transcription of repeat expansions has also limited the understanding of the role of antisense repeat RNAs. It remains challenging to weight the contribution of each transcript, sense or antisense, in the disease process. So far, it is known that some mutant antisense repeat transcripts can modulate gene transcription, dysregulate the activity of sense repeat transcripts or other complementary RNAs, sequester RBPs and be translated into toxic peptides. Intriguingly, a dysregulation of gene expression has arisen associated with antisense repeat transcription in at least four neurological diseases (Figure 2). Therefore, there is an emerging need to understand the mysteries behind the role of antisense repeat RNAs. Targeting both sense and antisense repeats may be required to improve the success of therapeutic strategies for these diseases.

## Figures and Tables

**Table 1:** Neurodegenerative and neuromuscular diseases with antisense repeat expansion transcription

Disease	Gene	Function of the Encoded Protein or RNA	Pathogenic Alleles	Proteins Sequestered in RNA foci	Proteins in Intranuclear Inclusions	Proteins in Cytoplasmic Stress Granules	Translated Repeat Polypeptides
SCA2	*ATXN2*	Endocytosis, translation, and mitochondrial function	(CAG)_>33_	ND	ND	STAU1 ^1^, FOX/A2BP1 ^1^, TDP-43 ^1^, PolyQ ^1^, ubiquitin ^1^, DDX6 ^2^, PABPC1 ^3^	ND *
	*ATXN2-AS*	Unknown	(CTG)_>33_	MBNL1 ^2^		ND	NF ^2^
SCA7	*ATXN7*	Assembly and maintenance of TFTC/STAGA complexes	(CAG)_>37_	ND	CBP ^3^, PolyQ ^1^, MBNL1 ^1^, pTDP-43 ^1^ and FUS/TLS ^1^, GCN5 ^2^	ND	ND
	*SCAANT1*	Transcriptional regulation of ATNX7	(CTG)_>37_		ND		
SCA8	*ATXN8*	Unknown	(CAG)_>80_	ND	PolyQ ^1^, ubiquitin ^1^	ND	PolyQ ^1^, PolyA ^1^, PolyS ^1^
	*ATXN8OS*		(CTG)_>80_	MBNL1 ^1^	ND		PolyL ^2^, PolyC ^2^, PolyA ^2^
SCA31	*BEAN1*	NEDD4-mediated ubiquitination	(TGGAA)_ins_	TDP-43 ^1^, FUS ^4^, hnRNPA2/B1 ^4^	ND	ND	PolyWNGME ^1^
	*SCA31AS*	Phosphorylation	(TTCCA)_ins_	NF ^1^			ND
FRDA	*FXN*	Iron metabolism	(GAA)_>66_	ND	ND	ND	ND
	*FAST-1*	Unknown	-				
SCA36	*NOP56*	Pre-rRNA processing	(TGGGCC)_>650_	SRSF2 ^1^	ND	ND	PolyGP ^1^, PolyWA ^2^, PolyGL ^2^
	*NOP56AS*	Unknown	(GGCCCA)_>650_	ND			PolyPR ^1^, PolyGP ^1^, PolyAQ ^2^,
*C9ORF72* FTD/ALS	*C9ORF72*	Regulation of endosomal trafficking	(GGGGCC)_>30_	SRSF1 ^1^, SRSF2 ^1^, ALYREF ^1^, ADARB2 ^1^, nucleolin^1^, Purα ^1^, hnRNPA2/B1 ^1^, hnRNPH ^1^, hnRNPF ^1^	ND	pTDP-43 ^1^, polyGR ^3^	PolyGA ^1^, PolyGR ^1^, PolyGP ^1^
	*C9ORF72AS*	Unknown	(CCCCGG)_>30_	SRSF2 ^1^, ALYREF ^1^, hnRNPA1 ^1^, hnRNPH/F ^1^, hnRNPK ^1^		ND	PolyPR ^1^, PolyPA ^1^, PolyGP ^1^
FXTAS	*FMR1*	mRNA trafficking from the nucleus to the cytoplasm	(CGG)_55–200_	Purα ^1^, hnRNPA2/B1 ^1^, Sam68 ^1^, CELF ^1^, MBNL1 ^1^, Rm62 ^1^, DGCR8 ^1^	SUMO2 ^1^, MLF2 ^1^, MBP ^1^, ubiquitin ^1^, p62 ^1^, hnRNPL ^1^, hnRNPA1 ^1^, hnRNPA3 ^1^, hnRNPC ^1^, U2AF ^1^, SFPQ ^1^ RAD50 ^1^, RPA1 ^1^, XRCC6 ^1^	ND	PolyG ^1^, PolyA ^2^
	*ASFMR1*	Unknown	(CCG)_55–200_	ND	ND	ND	PolyP ^1^, PolyA ^1^, PolyR^2^
NIID	*NOTCH2NLC*	Neuronal proliferation and differentiation	(CGG)_>66_	ND	ND	ND	ND
	*NOTCH2NLC-AS*	Unknown	(CCG)_>66_				
OPML1	*LOC642361*	Unknown	(CGG)_exp_	ND	ND	ND	ND
	*NUTM2B-AS2*	Unknown	(CCG)_exp_				
HD	*HTT*	Unknown	(CAG)_>36_	MBNL1 ^1^, Nucleolin ^3^	CBP ^1^, mSIN3a ^1^, TBP ^1^, PolyQ ^1^, ubiquitin ^1^, p53 ^3^	Caprin-1^2^, G3BP^2^	PolyQ ^1^, PolyA ^1^, PolyS ^1^
	*HTTAS1*	HTT regulation	(CTG)_>36_	ND	NA	ND	PolyC ^1^, PolyL ^1^, PolyA ^1^
HDL2	*JPH3*	Formation of junctional membrane complexes	(CTG)_>40_	MBNL1 ^1^	ND	ND	ND
	*HDL2AS*	Unknown	(CAG)_>40_	ND	CBP ^1^, PolyQ ^1^, ubiquitin ^1^		
DM1	*DMPK*	Serine-threonine kinase	(CTG)_>50_	MBNL1 ^1,5^, MBNL2 ^1,5^, MBNL3 ^5^, hnRNPH ^1^, hnRNPF ^1^, Proteosome subunits ^1^	ND	ND	PolyL ^2^, PolyC ^2^, PolyA ^2^
	*DMPKAS*	Regulation of *DMPK* expression	(CAG)_>50_	ND			PolyQ ^5^, PolyS ^2^, PolyA ^2^
DM2	*CNBP*	Nucleic acid-binding protein	(CCTG)_>75_	MBNL1 ^5^, MBNL2 ^5^, MBNL3 ^5^, RNA Pol II ^5^, CStF ^5^, PML ^5^	ND	ND	PolyLPAC ^1^
	*CNBPAS*	Unknown	(CAGG)_>75_	hnRNPA1 ^1^			PolyQAGR ^1^

ND not determined; NF not found; ^1^ human brain tissues and derived cells; ^2^ human transfected cell lines; ^3^ transgenic mouse brain tissues; ^4^ Drosophila neuronal tissues; ^5^ human muscle tissues and derived cells; * Slight expression of RAN-translated polyQ peptides in transfected HEK293T cells, expression of C-terminal tags under the human cytomegalovirus (CMV) promoter.
